# Use of NIR in COVID-19 Screening: Proof of Principles for Future Application

**DOI:** 10.1021/acsomega.4c06092

**Published:** 2024-10-01

**Authors:** Matthews S. Martins, Marcia H. C. Nascimento, Leonardo B. Leal, Wilson J. Cardoso, Vandack Nobre, Cecilia G. Ravetti, Paula Frizera Vassallo, Reinaldo F. Teófilo, Valerio G. Barauna

**Affiliations:** † Department of Physiological Sciences, Universidade Federal do Espírito Santo, Av. Mal. Campos, 1468 - Maruípe, Vitória, Espírito Santo 29047-105, Brazil; ‡ Department of Chemistry, Universidade Federal Espírito Santo, Av. Fernando Ferrari, 514 - Goiabeiras, Vitória, Espírito Santo 29075-910, Brazil; § Departament of Chemistry, Universidade Federal de Viçosa, Viçosa, Minas Gerais 36570-900, Brazil; ∥ Interdisciplinary Research Center in Intensive Medicine (NIIMI) and Department of Clinical Medicine, Universidade Federal de Minas Gerais (UFMG), Av. Prof. Alfredo Balena, 110 - Santa Efigênia, Belo Horizonte, Minas Gerais 30130-100, Brazil

## Abstract

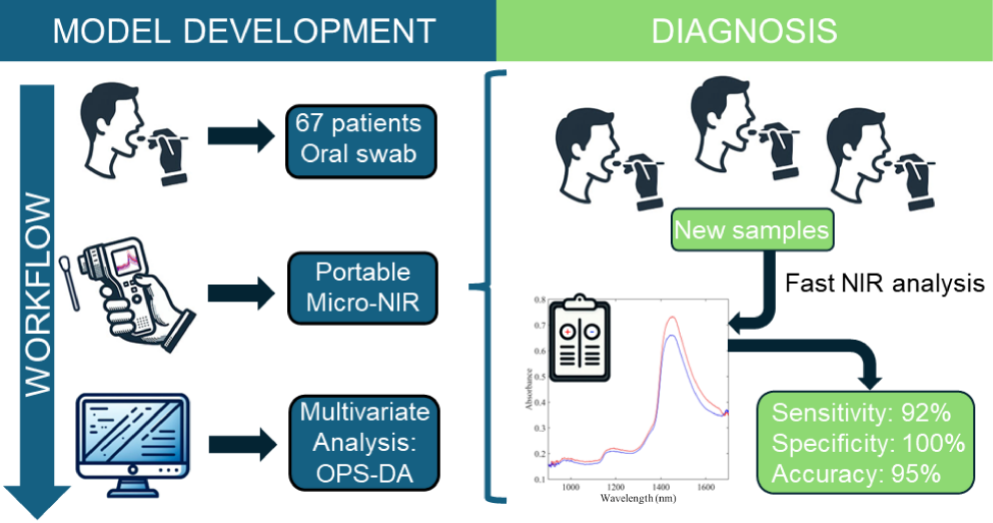

The COVID-19 pandemic that affected the world between 2019 and 2022 showed the need for new tools to be tested and developed to be applied in global emergencies. Although standard diagnostic tools exist, such as the reverse-transcription polymerase chain reaction (RT-PCR), these tools have shown severe limitations when mass application is required. Consequently, a pressing need remains to develop a rapid and efficient screening test to deliver reliable results. In this context, near-infrared spectroscopy (NIRS) is a fast and noninvasive vibrational technique capable of identifying the chemical composition of biofluids. This study aimed to develop a rapid NIRS testing methodology to identify individuals with COVID-19 through the spectral analysis of swabs collected from the oral cavity. Swab samples from 67 hospitalized individuals were analyzed using NIR equipment. The spectra were preprocessed, outliers were removed, and classification models were constructed using partial least-squares for discriminant analysis (PLS-DA). Two models were developed: one with all the original variables and another with a limited number of variables selected using ordered predictors selection (OPS-DA). The OPS-DA model effectively reduced the number of redundant variables, thereby improving the diagnostic metrics. The model achieved a sensitivity of 92%, a specificity of 100%, an accuracy of 95%, and an AUROC of 94% for positive samples. These preliminary results suggest that NIRS could be a potential tool for future clinical application. A fast methodology for COVID-19 detection would facilitate medical diagnoses and laboratory routines, helping to ensure appropriate treatment.

## Introduction

At the end of 2019, a new strain of coronavirus, designated as severe acute respiratory syndrome coronavirus (SARS-CoV-2), emerged in Wuhan, China, leading to an unusual respiratory illness.^1^ This novel disease, called coronavirus disease 2019 (COVID-19), has rapidly spread worldwide due to its high transmissibility via respiratory droplets and contact routes.^2^

Depending on patient-specific factors, individuals with COVID-19 may progress to severe illness, including acute respiratory distress syndrome (ARDS), multiple organ failure, and potentially death, especially in older groups and patients with comorbidities.^3,4^ Given this scenario, the application of diagnostic testing and the rapid use of the results remains fundamental to identifying infected people and implementing an appropriate therapy, even after global immunization programs.^5^

SARS-CoV-2 RNA is still detected via reverse-transcription polymerase chain reaction (RT-PCR), most commonly collected from nasopharyngeal swabs.^6^ Although RT-PCR is considered the gold standard for diagnosis, its high cost, lengthy result times, and extensive sample preparation pose challenges for mass testing.^7,8^ Furthermore, the correct procedure is crucial for the sensitivity and specificity of the assays.^9^

Vibrational spectroscopy has attracted growing interest due to its ability to discriminate and classify normal and pathological patients through the vibrational modes of specific functional groups in lipids, proteins, and nucleic acids.^10^ Among vibrational spectroscopic techniques, mid-infrared (MIR) and near-infrared (NIR) spectroscopy have been widely used due to their simplicity, reproducibility, speed, and nondestructive nature for the analyzed sample.^11,12^

The NIR region in the electromagnetic spectrum lies between the visible and mid-infrared regions. NIR radiation interacts with samples, causing their molecular structures to vibrate as they absorb part of the radiation, which allows for their identification. This technique becomes a powerful tool when coupled with a multivariate analysis approach. It can explore and detect changes in the biomolecular content of healthy and diseased tissues by assigning functional groups based on spectral information.^13,14^

As a result, NIR spectroscopy has immense potential in detecting human diseases, including bacterial and viral infections.^15^ Therefore, this study aimed to develop a rapid and noninvasive NIR spectroscopic testing methodology and evaluate its potential for future clinical applications. This methodology aims to identify individuals with COVID-19 using oral swab samples based on the analysis of spectral chemical information.

## Methods

### Participants and Samples

In this study, we evaluated NIR spectra from 67 individuals hospitalized in either the intensive care unit (ICU) or hospital ward of the University Hospital at the Federal University of Minas Gerais (UFMG) with clinical suspicion of having COVID-19 between June 2020 and September of 2020. The Federal University of Espírito Santo Ethics Committee granted ethical approval for the investigation (0993920.1.0000.5071 and 31411420.90000.8207). All individuals provided the Informed Consent Form. Participant characteristics were compiled, including comorbidities, disease severity, and hospitalization information from the medical records.

All diagnoses were given by RT-PCR analyses, resulting in positive (*n* = 46) and negative results (*n* = 21). Samples were collected using a synthetic fiber-tipped swab (rayon), as cotton swabs could negatively affect virus detection by RT-PCR.^16^ The swab was scraped on both sides of the subject’s oral cavity and immediately taken for reading on NIRScan.

### NIR Spectral Acquisition

The equipment utilized for the analysis was a portable instrument, the DLP NIRscan Nano EVM (Texas Instruments). Using the Hadamard scan method, this instrument operates in the 900 to 1700 nm range with increments of 1.32 nm.

All swab samples were directly placed on the crystal of the NIRscan equipment, maintaining contact during scans. The swab was analyzed in three different positions for each patient, and the results were then averaged. After collecting each patient’s spectra, the equipment was cleaned with 70% alcohol and internal background was applied. The primary function of the background is to account for any changes in atmospheric or instrument conditions, preventing the previous measurement from influencing the next.^17^ Spectra were collected in triplicate across the full wavelength range of the equipment.

### Spectral Analysis and Outlier Removal

The average spectra were organized in an X (n,m) matrix, with *n* = 67 (samples) and *m* = 605 (variables). First, a Principal Components Analysis (PCA) was conducted to observe the spectra distribution and detect any potential anomalous samples. All calculations were performed in Matlab R2020a (Math Works, Natick, USA).

Principal Component Analysis (PCA) is commonly used to reduce the dimensionality of the original data, where the principal components (PCs) represent linear combinations of variables. Each sample can be represented by a few values and plotted in a reduced-dimension space (score plot).^18^ It allows for recognizing similarities and differences among samples by observing their distribution.^19^

Well-established standardization protocols for biological spectrochemical data sets indicate that quality evaluation of spectra should be performed before preprocessing.^20,21^ This can be achieved through principal component analysis (PCA) to identify anomalous samples due to biased experimental measurements. After performing PCA, seven outliers were identified and removed (3 positive and 4 negative).

### Classification Analysis and Variable Selection

Partial Least Squares for Discriminant Analysis (PLS-DA) was employed to construct classification models for two distinct classes: COVID-19 positive and COVID-19 negative. These classifications were determined using RT-PCR results. The data (*n* = 60) was split into training (70%) and testing (30%) sets using the Kennard-Stone algorithm.^22^

Spectra of both sets were preprocessed using the first derivate (window: 3, polynomial order: 2) and normalization using Standard Normal Variate (SNV). Random cross-validation was applied, where splits were set at 10% of the training set.

PLS-DA is a widely used linear two-class classifier method (PLS1 algorithm). This algorithm determines the group to which a sample belongs using its spectral profile.^23^ A model is developed based on the correlation between the training set matrix and the y vector, which indicates the classes to which the samples belong (class 1, positive; class 0, negative). Subsequently, the PLS-DA model performs binary discrimination, categorizing the samples into one of the pre-established classes.^24^

Variable selection in PLS-DA models was conducted using Ordered Predictors Selection (OPS),^26,27^ adapted for classification models (OPSDA), to identify more interpretative variables and enhance the classification model. As a result, two models were obtained: (i) PLS-DA using all variables (Full), and (ii) PLS-DA with variable selection via OPS (OPSDA).

Classification models were evaluated using parameters such as sensitivity (eq 1), specificity (eq 2), classification error (eq 3), and Matthews’s correlation coefficient (MCC) (eq 4). Sensitivity is the percentage of samples of each class accepted by the class model. Specificity is the percentage of samples of the other class correctly rejected by the class model. Classification error is the percentage of samples classified in the wrong class.^28^ MCC is a classification metric that evaluates prediction by considering positive and negative data instances. In ranges from −1 (perfect misclassification) to +1 (perfect classification).^29^
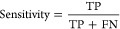
1
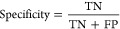
2
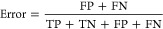
3
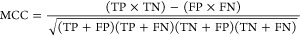
4

Where TP is a true positive (number of samples that belong to the class classified as belonging to class), FN is a false negative (number of samples that belong to the class not classified as belonging to class), TN is true negative (number of samples that do not belong to the class classified as not belonging to class), and FP is false positive (number of samples that do not belong to the class classified as belonging to class).

These sensitivity and specificity values were also utilized to construct the Receiver Operating Characteristic (ROC) curve. A ROC plot might be viewed as the mean sensitivity of a test across all conceivable specificity values or conversely. Additionally, the area under the ROC curve (AUC) is an effective way to observe the overall accuracy of the test.^28^

### Statistical Analysis

The statistical analyses between groups (COVID-19 positive and negative) were determined by either the chi-square test or Fisher’s exact test for qualitative variables and either the *t* test or Mann–Whitney test for quantitative variables, depending on whether the distribution was parametric or nonparametric, respectively. The confidence interval was set at 95%. The difference was considered statistically significant when *p* < 0.05.

## Results and Discussion

Table 1 describes participant characteristics by age, gender (male), comorbidities, severity, and hospitalization information. There was no statistical difference in the clinical profile between the patients with or without COVID-19. The patients positive for COVID-19 stayed more days hospitalized in the ICU (*p* = 0.004), and even though ICU mortality was not statistically different, *p* = 0.06, a trend of higher mortality was observed in these patients.

**Table 1:** Health Data for Participants^a^

		Total (*n* = 67)	Positive (*n* = 45)	Negative (*n* = 22)	p-value
age (years)	median (range)	65 (52–74)	64 (51–73)	66 (54–75)	0.738
gender	male (%)	37 (55.2%)	26 (57.8%)	11 (50.0%)	0.548
comorbidities	HAS (%)	40 (59.7%)	29 (64.4%)	11 (50.0%)	0.258
	DM (%)	24 (35.8%)	18 (40.0%)	6 (27.3%)	0.308
	COPD (%)	12 (17.9%)	6 (13.3%)	6 (27.3%)	0.187
	Asthma (%)	3 (4.5%)	2 (4.4%)	1 (4.5%)	1.000
	Obesity (%)	12 (17.9%)	9 (20.0%)	3 (13.6%)	0.737
severity	SOFA (range)	7 (3–10)	6 (3–9)	7,5 (4.7–11)	0.192
admitted to ICU	number of patients (%)	62 (92.5%)	41 (91.1%)	21 (95.5%)	1.000
days hospitalized	ICU—median (range)	9 (4–12)	10 (6.5–19.2)	4 (3–7)	**0.004**
	hospital—median (range)	20 (11–35)	23 (11–34)	19 (9–51)	0.867
death	ICU (%)	31 (50.0%)	24 (58.5%)	7 (33.3%)	0.060
	Hospital (%)	34 (50.7%)	26 (57.8%)	8 (36.4%)	0.100

a HAS = hypertension; DM = diabetes mellitus; COPD = chronic obstructive pulmonary disease; SOFA = Sequential Sepsis-related Organ Failure Assessment; ICU = intensive care unit —confidence interval of 95%.

A prolonged hospital stay in patients with COVID-19 was also observed in other studies.^30−32^ Clinical complications could explain this finding in patients with severe COVID-19, like acute respiratory distress syndrome (ARDS), acute kidney injury (AKI), delirium/encephalopathy, cardiac injury (e.g., cardiomyopathy, arrhythmia, and sudden cardiac death), thrombosis, secondary hospital infection. All these clinical conditions impact the duration of hospitalization.^33−37^

Also, mechanical ventilation is prolonged in patients with COVID-19-related ARDS compared with non-COVID-19-related ARDS, with many COVID-19 patients remaining intubated for at least one to 2 weeks or longer.^32^

The Sequential Organ Failure Assessment (SOFA) is today’s most used score in general ICUs. It evaluates respiratory, hematological, hepatic, cardiovascular and neurological function. A score of 3 or 4 for each function indicates organic failure, and failure of 3 or more organs/systems results in a mortality rate greater than 70%.^38,39^ The patients in this study presented clinical severity as indicated by the median SOFA score of 7.

In critically ill patients, a diagnostic method that is fast and easy to collect and analyze impacts daily care since clinical instability and severity could be a major challenge. Also, early diagnosis of diseases that require respiratory and contact isolation is of primary importance for the optimization of the distribution of hospital beds, especially in the case of epidemics or pandemics.

### Spectral Analysis

Spectra were collected using portable NIRscan equipment from 900 to 1700 nm, a subset of the full NIR range (750–2500 nm).^40^ It is a typical limitation observed in portable instruments, mainly due to design constraints, as the instrument is very compact and designed to be carried everywhere. Despite this limitation, the spectral information captured was sufficient for sample discrimination after selecting the most informative variables.

Another topic of discussion is the spectral interference caused by the rayon swab, which could suppress information about biomolecules. Rayon is a synthetic fiber mainly composed of cellulose. The significant bands observed in rayon NIR spectra are around 1500 nm (O–H stretch overtone), 2000 nm (combination band region), and 2100 nm (C–O stretch overtone).^41^ The variables selected by OPSDA, considered the most discriminative, were located in other regions related to the biological samples.

**Figure 1:**
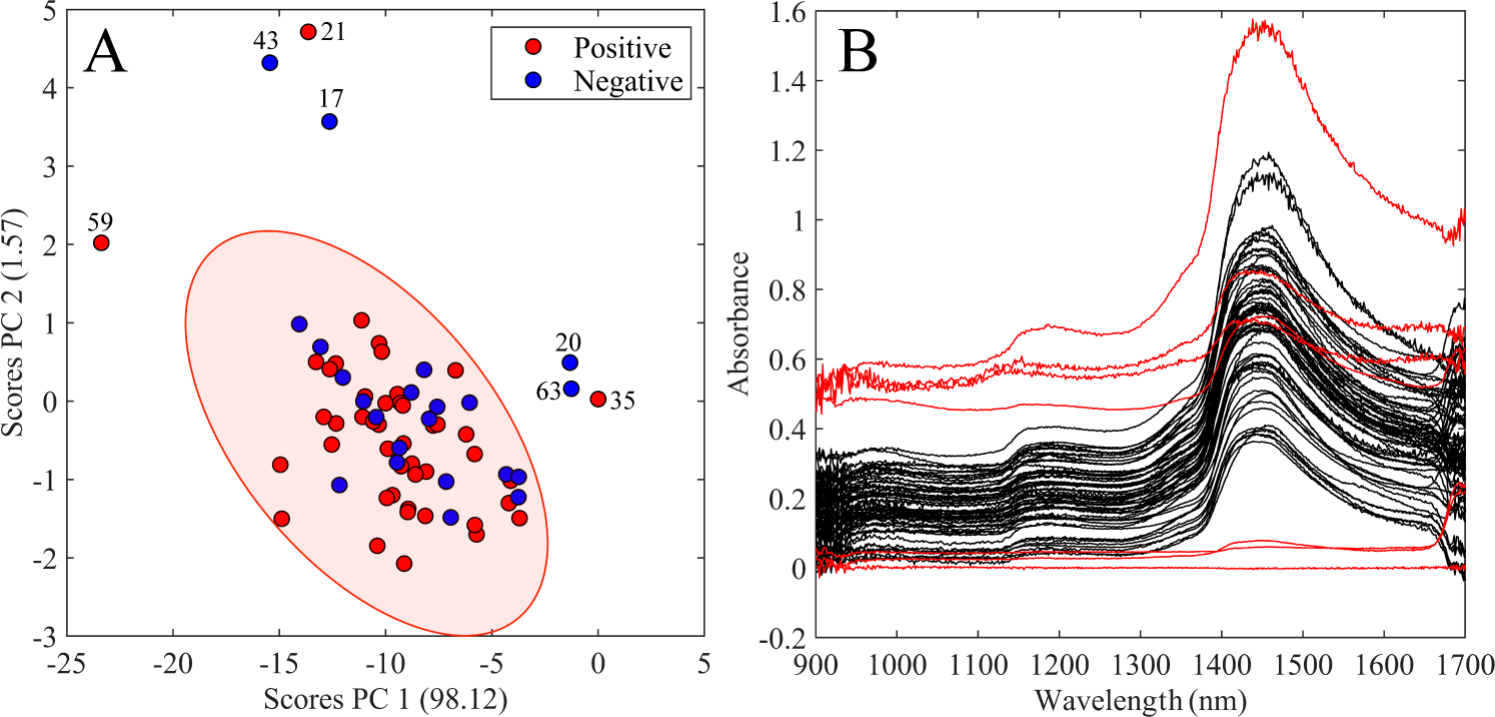
A) Score plot for Principal Component Analysis for all samples (PC1 X PC2). Red dots represent the positive and blue dots represent the negative samples. The ellipse shows a confidence interval of 95%. B) NIR spectra of the samples. Red lines represent the outliers removed, and black lines are the samples kept for modeling.

To identify anomalous samples due to biased experimental measurements, raw spectra were projected in two dimensions by the PCA scores graph (Figure 1A), with a 95% confidence interval ellipse. Samples located outside the ellipse were identified as outliers. Subsequently, the 4 negative and the 3 positive outliers were spotted in the group of spectra (Figure 1B) and then removed since they presented an anomalous spectral profile.

**Figure 2:**
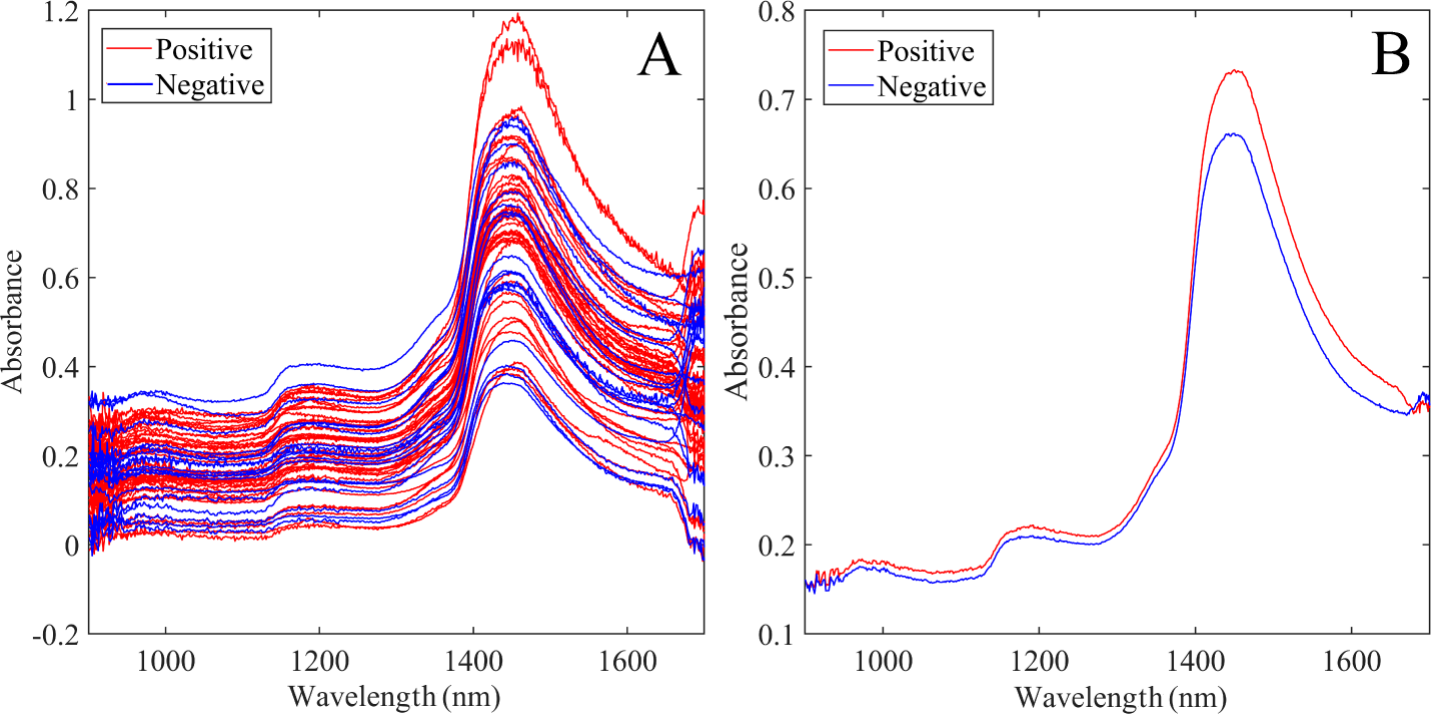
A) All raw NIR spectra after outlier removal. Blue lines represent negative samples, and red lines represent positive samples. B) Mean of positive (red) and negative (blue) groups from raw NIR spectra after outlier removal.

Figure 2 displays raw spectra profiles after outlier removal, distinguishing positives (red) and negatives (blue) for COVID-19. Additionally, the mean spectra of both groups can be observed in Figure 2B, showing greater intensity of the positive group bands.

### Classification Analysis

After outlier removal, the models were constructed with 60 samples (42 positives and 18 negatives for COVID-19). The results of the two models built with preprocessed spectra are presented in Table 2: the full model is the PLS-DA using all variables, while the OPSDA is the PLS-DA with variable selection via OPS.

**Table 2:** Diagnostic Metrics for the Model Using all Variables (Full) and Selected Variables (OPSDA)

	Full			
	Training (%)		Test (%)	
	Negative	Positive	Negative	Positive
**Error**	0	0	44	44
**Sensitivity**	100	100	60	54
**Specificity**	100	100	54	60

	OPSDA			
	Training (%)		Test (%)	
	Negative	Positive	Negative	Positive
**Error**	2	2	5	5
**Sensitivity**	100	96	100	92
**Specificity**	96	100	92	100

For the OPSDA model, 20 variables were selected, decreasing variable collinearity. The variables with the most importance for group separation were 903, 908, 915, 916, 918, 934, 957, 968, 1006, 1012, 1102, 1142, 1422, 1428, 1569, 1621, 1670, 1675, 1698, and 1700 nm. Wavelengths at 970 nm and around 1400 nm (O–H stretch overtone) are related to water and were previously demonstrated as important discriminant variables in studies of other viral diseases using NIR, such as HIV-1, influenza, ZIKV and CHIKV. This phenomenon might be due to reduced water molecule components as viral concentrations increase.^40^ Furthermore, the significant bands associated with rayon were not selected by OPSDA, as discussed.

Table 2 compares the two models developed in this study, which were constructed using six latent variables. The first model was built with all variables (full) obtained from the analysis of vibrational spectroscopy using NIR. In this initial model, no error was obtained in the model calibration. However, when tested with a blind test, there was an increase in the prediction error for these samples, with a decreased sensitivity of 54% and specificity of 60% for the positive class, resulting in an accuracy of 57%.

For the OPSDA model, a sensitivity of 96% and specificity of 100% were achieved for a truly positive class during calibration. In the test set, the results surpassed the model with all variables, achieving a sensitivity of 92% and specificity of 100% for positive class, resulting in a 95% accuracy for the correct diagnosis of COVID-19.

The ratio of positive to negative COVID-19 patients in the study is unbalanced (42:18), reflecting the actual proportion of infected patients at the time of sample collection. Balanced classes are preferred in classification models to avoid bias toward the majority class, the positive group. However, the test set evaluation for the OPSDA model showed a lower sensitivity in the positive group (92%) when compared to the negative group (100%), which is the opposite of what would be expected if there was an overfitting due to unbalanced classes. It strongly indicates that OPSDA effectively identified key features related to both positive and negative samples.

The decreased performance in the full model for test set evaluation may be associated with the collinearity problem, where redundant information causes interference in the modeling by inflating the variance.^42,43^ In this case, a simple variable selection method was sufficient to reduce the number of correlated variables and select those of major importance, thereby enhancing the model’s performance.

In Table 3, the contingency matrix for the calibration and prediction sets of the OPSDA model can be observed, demonstrating a significant ability to identify truly positive and truly negative samples. 41 out of 42 samples were correctly predicted in calibration and 17 out of 18 correctly predicted in the blind-test.

**Table 3:** Contingency Matrix of the Training Set and Test Set

		Reference			
		Training		Test	
		Negative	Positive	Negative	Positive
**Predicted**	**Negative**	13	1	5	1
	**Positive**	0	28	0	12

The Matthews correlation coefficient (MCC) was calculated using information from the contingency matrix. This diagnostic metric has proven to be a reliable statistical measure, as it is unaffected by unbalanced data sets. It is the only classification rate that generates high scores if the prediction is correct for most positive and negative cases.^29^ The MCC for the OPSDA model test set was 0.87, indicating good predictions in both classes and demonstrating no overfitting despite the unbalanced data sets.

**Figure 3:**
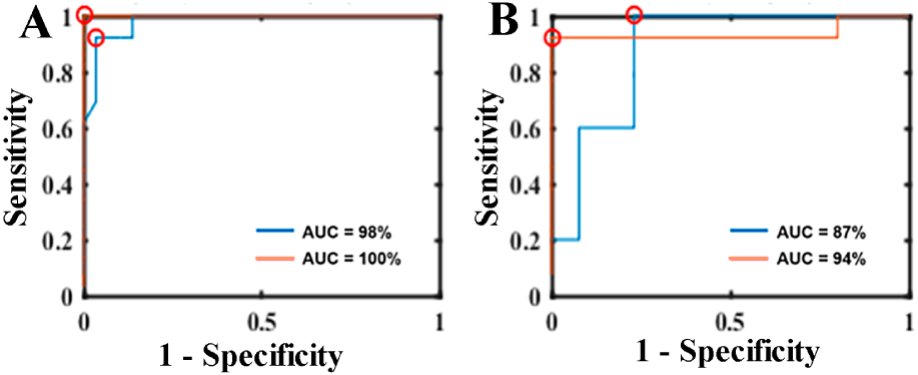
A) Receiver Operating Characteristic (ROC) curve with sensitivity and specificity from the calibration set. B) ROC curve with sensitivity and specificity from the prediction set. The blue line represents negative samples, and the orange line represents positive samples.

In Figure 3A (calibration set) and 3B (prediction set), the ROC curve of the model is depicted for the negative (blue) and the positive (orange) samples. It shows an excellent AUC result for prediction (blind test), reaching 87% for the negative and 94% for the positive samples.

Several authors have employed near-infrared spectroscopy to diagnose and detect viruses such as HIV, hepatitis, influenza, and Zika.^44−47^ Most studies using spectroscopy to detect COVID-19 use mid-infrared (MIR) wavelengths. However, NIR is less expensive than MIR techniques, and its radiation penetrates farther into samples due to its higher energy.^40^

Coelho et al.^48^ tested the feasibility of visible and NIR (Vis-NIR) spectroscopy to detect individuals with COVID-19 using nasopharyngeal swab samples. The authors achieved an accuracy of 75%, a sensitivity of 80% and a specificity of 70%. However, the swab was not directly analyzed; the viral transport liquid was used, requiring additional sample preparation. Additionally, despite the portable equipment, the approaches required an external lamp or a white reflexive screen. These extra steps could impact cost-effectiveness and the speed of analysis.

Some authors have achieved similar metrics to ours using MIR for its detection in saliva. Wood et al.^49^ found a sensitivity of 93% and a specificity of 83% for the positive class, with 27 out of 29 samples correctly classified as the disease group. Nascimento et al.^50^ achieved a sensitivity of 93%, a specificity of 83%, and an accuracy of 85% using unsupervised random forest models. In both cases, the equipment used for spectra collection was not portable, and sample preparation was necessary.

Based on these preliminary results obtained, we can assert that NIR spectroscopy, when combined with multivariate data analysis such as OPSDA, holds significant potential for aiding in the detection of patients suspected of having COVID-19 using swabs collected directly from the oral cavity. These samples can be collected noninvasively and quickly, facilitating laboratory routines and medical diagnoses. In critically ill patients, a screening method that is fast and easy to collect impacts daily care since the clinical instability sometimes is a challenge in collecting samples.

## Conclusion

After years of the COVID-19 pandemic, an efficient screening test that provides truly reliable, fast, and cost-effective results is still necessary. For this purpose, we conducted a proof-of-concept study to develop a NIR spectroscopic testing methodology for detecting individuals with COVID-19 using oral swabs and evaluated its potential for future clinical application. The tests conducted with the proposed OPSDA model (*n* = 60) have proven effective. The model achieved a sensitivity of 92%, a specificity of 100% and an accuracy of 95% in test set validation. Regarding the ROC curve, the AUC was 94% for positive samples. This preliminary data indicates that this methodology has significant potential as a screening tool in clinical and hospital settings. However, further analysis with more samples and multicentric collection should be conducted for more appropriate external validation. Finally, the many benefits of this technique’s application in clinical routines include reduced resource demand, noninvasive sample collection, fast analysis and the facilitation of medical diagnoses.
